# A Decade of *GigaScience*: The Challenges of Gigapixel Pathology Images

**DOI:** 10.1093/gigascience/giac056

**Published:** 2022-06-14

**Authors:** Geert Litjens, Francesco Ciompi, Jeroen van der Laak

**Affiliations:** Department of Pathology, Radboud University Medical Center, Nijmegen, The Netherlands; Department of Pathology, Radboud University Medical Center, Nijmegen, The Netherlands; Department of Pathology, Radboud University Medical Center, Nijmegen, The Netherlands; Center for Medical Image Science and Visualization, Linköping University, Linköping, Sweden

## Abstract

In the last decade, the field of computational pathology has advanced at a rapid pace because of the availability of deep neural networks, which achieved their first successes in computer vision tasks in 2012. An important driver for the progress of the field were public competitions, so called ‘Grand Challenges’, in which increasingly large data sets were offered to the public to solve clinically relevant tasks. Going from the first Pathology challenges, which had data obtained from 23 patients, to current challenges sharing data of thousands of patients, performance of developed deep learning solutions has reached (and sometimes surpassed) the level of experienced pathologists for specific tasks. We expect future challenges to broaden the horizon, for instance by combining data from radiology, pathology and tumor genetics, and to extract prognostic and predictive information independent of currently used grading schemes.

## Background

Not only was 2012 the year in which the first volume of *GigaScience* was published, but for many in the computer vision community it was the year that deep convolutional neural networks (CNN) revolutionized the field. In December 2012, the amazing results of Alex Krizhevsky and colleagues on the ImageNet Large Scale Visual Recognition Challenge were presented at the NeurIPS conference [1]. What most people do not realize, however, is that this was not the first competition in which CNNs outperformed traditional image analysis methods by a large margin. At the ICPR conference in November of 2012, the contest on Mitosis Detection in Breast Cancer Histological Images was won by Dan Ciresan and colleagues, using a strategy that was very similar to the one of Krizhevsky and colleagues a month later [2]. These events mark the beginning of a massively increased interest in the field of computational pathology (CPATH), in which datasets, methods and applications started to grow, develop and diversify at a rapid pace.

## Main Text

Even though the ICPR2012 (and the subsequent AMIDA13 mitosis counting challenge at the MICCAI conference a year later) were important stepping stones, achieving clinical impact was not yet feasible. Dataset sizes were still relatively small, with AMIDA13, for instance, only consisting of images obtained from 23 patients in total, and solely limited to smaller fields of view, up to 2048 pixels in width and height. These are not representative of entirely scanned tissue sections (whole-slide images; WSI), which are routinely used in digital pathology and can be gigapixels in size.

In a pursuit to reach clinical impact in Pathology practice, in 2014 we started to study deep learning-based methods that would be directly applicable to WSI. To be able to deal with the heterogeneity typically present in and between real-world pathology images, we used much larger datasets. This led to one of the first publications on the application of CNNs to WSI, in the context of two clinically relevant tasks: detection of prostate cancer in biopsy specimens, and of breast cancer metastases in lymph node tissue sections [3]. The latter task is highly relevant for breast cancer staging: pathologists need to identify, within millions of cells, whether there are, even very small, clusters of metastatic cancer cells. Given its tedious and time-consuming nature, this task bears a significant risk of missing small tumor cell clusters (so-called micro-metastases), an ideal test case to study the applicability of CNNs for clinically relevant diagnostic tasks in Pathology. Even though our initial results were promising, we decided to scale up our studies to achieve real-world impact: we organized the CAMELYON (CAncer MEtastases in LYmph nOdes challeNge) challenges, inviting researchers worldwide to aid in finding solutions for this task [4, 5].

In our initial study, we trained a CNN for metastases detection using 271 slides originating from a single institution. We realized that across different institutions, our algorithm would underperform because of commonly present variations in tissue fixation, processing, staining and scanning as well as from other sources influencing image appearance. We therefore extended the dataset significantly, involving other centers and increasing diversity of the data. In 2016, this resulted in organizing the CAMELYON16 challenge at the ISBI2016 conference, using a dataset of 400 WSI from our own lab and from the University Medical Center in Utrecht, the Netherlands [4]. Subsequently, at the ISBI2017 conference we presented results of CAMELYON17, which used a dataset of 1400 slides across five different Dutch centers [5]. At that time, this was the single largest public labeled WSI dataset. The results of the CAMELYON challenges were among the first to show the possibility of reaching, and even surpassing, human expert performance with CNNs for tasks in Pathology. These results were therefore highlighted in many publications, also outside of the scientific community (e.g., the White House Report on AI, Automation and the Economy). Additionally, the CAMELYON challenges led to increased commercial interest for CPATH, with the 2016 challenge-winner establishing the company Path. AI and Google working with the challenge data as their first foray into CPATH [6, 7].

Organizing these competitions has allowed us to better understand the challenges associated with sharing large datasets publicly with a large number of participants. With CAMELYON17, the main problem was the large data size of over 3 Terabytes, and the compute required to train competitive CNNs, causing a significant barrier and excluding many groups from participating who did not have access to sufficiently powerful compute facilities. Probably as impactful, though not anticipated beforehand, were difficulties associated with the accessibility of data in certain geographical areas. We initially shared challenge data using a cloud provider to handle the massive load but did not realize that it was not accessible in, for example, China, requiring us to use BaiduPan as a mirror. WSI are also difficult to work with, while at the time of the CAMELYON challenges there were very few tutorials on how to use open-source tools effectively for this purpose. This was one of the key reasons we decided to publish the CAMELYON dataset, including descriptions of use, in *GigaScience*, sharing the data through GigaDB [7]. Another problem we ran into while organizing the CAMELYON challenges: we publicly released the test set images, without the labels. However, it later became apparent this still carries a risk of overfitting to the test set, even if the evaluation is done independently as pseudo-labeling.

With the finalization of the CAMELYON challenges in 2018, it became clear that many cancer detection and segmentation tasks in histopathology could be solved with well-curated datasets and state-of-the-art computer vision methods. The focus therefore shifted to a more advanced stage of CPATH: *prognostication*. For many cancer types, pathologists play a key role in providing the information on which treatment decisions are based, by visual inspection of tissue morphology and assessing the degree (‘grade’) of aggressiveness of the tumor. Higher grades are typically associated with a worse prognosis for the patient. Overall, grading schemes work reasonably well within various different types of cancer, such as prostate or breast cancer, but suffer from inter- and intra-observer variability. This offers two distinct avenues for machine learning algorithms: 1) to learn to replicate the grading scheme and offer more reliable, quantitative grading, 2) to learn to directly predict patient prognosis from tissue morphology using digital biomarkers.

Much research has been devoted to the development of Deep Learning for these two directions. We have approached these through two new public challenges, including large data sets. While doing so, we managed to solve some of the shortcomings we encountered previously. Together with the Karolinska Institute and Google Health, we organized the PANDA (Prostate cANcer graDe Assessment) challenge for prostate cancer grading, using biopsy data from over 6000 patients [8]. Participants had to predict a consensus grade (established by a panel of experienced pathologists) based on only a biopsy WSI, without any detailed pixel-level annotations. To stimulate participation of researchers in less resourceful conditions, we spent ample time on data preparation: identifying the best WSI compression rate, removing intermediate resolution levels from the image files, removing consecutive sections of the same biopsy, etc. We hosted the challenge via Kaggle, allowing participants to run their solutions through a Jupyter Notebook interface without ever downloading the data. The test set was kept hidden from participants, but rather they submitted their algorithms which were ran on the test cases by us. Therewith, overfitting on the test set was prevented. Again, the top algorithms in PANDA performed at the level of experienced pathologists.

For the second direction, together with the international Immuno-Oncology Working Group, we recently initiated the TIGER (Tumor InfiltratinG lymphocytes in breast cancER) challenge in which participants try to improve prediction of breast cancer prognosis. In TIGER, participants train CNNs to detect tumor infiltrating lymphocytes (TILs) [9] and, for the first time in a public challenge, use that to predict the recurrence-free survival on a dataset of 707 patients with breast cancer. Like PANDA, participants submit their algorithms to the grand-challenge.org platform in the form of a Docker container, which we run on a hidden test set to prevent overfitting. This setup allowed us to validate submissions on sensitive data from clinical trials, which cannot legally be shared. This challenge is therefore a key example of how to still be able to use sensitive data in a public competition.

## Conclusions

Over the past decade, the CPATH field has moved from small datasets to large datasets and challenges to answer complex clinical questions ranging from cancer detection to grading and prognosis. However, for the next decade there is still a substantial amount of work to be done. For example, despite the vast amounts of data used in current studies, often these are still selected by a few researchers from a handful of centers. Data diversity can be vastly increased to better represent daily clinical practice [10]. One can imagine truly federated studies across many centers to prevent the limitations caused by anonymization and data sharing agreements.

Secondly, pathology is not a specialism in a vacuum: there are many complementary specialties (e.g., radiology, genetics) that provide additional information for patient management. As such, we also see cross-specialty studies as a meaningful path forward. Last, cancer prognostics was already an important avenue to address with large datasets and machine learning techniques, but treatment decision making is still an unexplored avenue, especially due to the challenging data collection (e.g., diversity in treatment trajectories, matching requirements, among others). Summarizing, the CPATH community has made a massive leap forward over the past decade, but there is much more still to come.

## Editor's Note

This commentary is part of a series to celebrate a Decade of *GigaScience*, to coincide with the 10th anniversary of our launch in July 2012. These papers take a look back at 10 years of advances in large-scale research as open science has become mainstream.

## Abbreviations

CAMELYON: CAncer MEtastases in LYmph nOdes challeNge; CNN: convolutional neural networks; CPATH: computational pathology; PANDA: Prostate cANcer graDe Assessment; TIGER: Tumor InfiltratinG lymphocytes in breast cancER; WSI: whole-slide images

## Data Availability

Not Applicable

## Competing interests

All reported interests below concern the last five years, and are outside the submitted work. G.L. reports grants from Philips Digital Pathology Solutions and personal fees from Novartis. F.C. was member of the Scientific and Medical Advisory Board of TRIBVN Healthcare (Paris, France). J.v.d.L. was a member of the advisory boards of Philips, the Netherlands and ContextVision, Sweden, and received research funding from Philips, the Netherlands, ContextVision, Sweden, and Sectra, Sweden. J.v.d.L. is chief scientific officer (CSO) of Aiosyn BV, the Netherlands. G.L., F.C. and J.v.d.L. are shareholders of Aiosyn BV, the Netherlands.

## Funding

G.L. acknowledges funding from the Dutch Cancer Society (KUN 2015-7970) and the Netherlands Organization for Scientific Research (NWO; project number 016.186.152). F.C. acknowledges funding from the European Union's Horizon 2020 research and innovation program under grant agreement no. 825292 (ExaMode, http://www.examode.eu/); the Dutch Cancer Society (KWF; project no. 11917); and the Netherlands Organization for Scientific Research (NWO; project no. 18388). J.v.d.L. acknowledges funding from the Knut and Alice Wallenberg Foundation, Sweden, and received funding from the Innovative Medicines Initiative 2 Joint Undertaking under grant agreement no. 945358. This Joint Undertaking receives support from the European Union's Horizon 2020 research and innovation program and EFPIA.

## Author contributions

All authors were involved in identifying relevant literature, and in drafting and revising the manuscript.
